# Mouse Tafazzin Is Required for Male Germ Cell Meiosis and Spermatogenesis

**DOI:** 10.1371/journal.pone.0131066

**Published:** 2015-06-26

**Authors:** Laurence C. Cadalbert, Farah Naz Ghaffar, David Stevenson, Sheila Bryson, Frédéric M. Vaz, Eyal Gottlieb, Douglas Strathdee

**Affiliations:** 1 Transgenic Technology Laboratory, Cancer Research UK Beatson Institute, Garscube Estate, Switchback Road, Glasgow, G61 1BD, United Kingdom; 2 Cell Death and Metabolism Laboratory, Cancer Research UK Beatson Institute, Garscube Estate, Switchback Road, Glasgow, G61 1BD, United Kingdom; 3 Laboratory Genetic Metabolic Diseases, Academic Medical Center, University of Amsterdam, Amsterdam, The Netherlands; Cardiff University, UNITED KINGDOM

## Abstract

Barth syndrome is an X-linked mitochondrial disease, symptoms of which include neutropenia and cardiac myopathy. These symptoms are the most significant clinical consequences of a disease, which is increasingly recognised to have a variable presentation. Mutation in the Taz gene in Xq28 is thought to be responsible for the condition, by altering mitochondrial lipid content and mitochondrial function. Male chimeras carrying a targeted mutation of Taz on their X-chromosome were infertile. Testes from the Taz knockout chimeras were smaller than their control counterparts and this was associated with a disruption of the progression of spermatocytes through meiosis to spermiogenesis. Taz knockout ES cells also showed a defect when differentiated to germ cells *in vitro*. Mutant spermatocytes failed to progress past the pachytene stage of meiosis and had higher levels of DNA double strand damage and increased levels of endogenous retrotransposon activity. Altogether these data revealed a novel role for Taz in helping to maintain genome integrity in meiosis and facilitating germ cell differentiation. We have unravelled a novel function for the Taz protein, which should contribute to an understanding of how a disruption of the Taz gene results in the complex symptoms underlying Barth Syndrome.

## Introduction

Barth syndrome (BTHS) is a rare X linked condition affecting young males. Patients typically display skeletal and cardiac myopathies, growth retardation and neutropenia, which often lead to an early death [1, 2]. Progress in managing heart defects and compromised immunity has significantly increased longevity for those afflicted [3] allowing them to live well into adulthood. BTHS is attributed to various mutations within the Taz gene at the Xq28 locus [4, 5]. Taz is a mitochondrial transacylase that regulates the production of mature cardiolipin [6, 7]. Cardiolipin is a lipid exclusively found in the mitochondrial membrane and which has been shown to anchor components of the respiratory chain within the mitochondrial membrane [8–10]. More recently, reports have suggested a role for Tafazzin in membrane remodelling [11, 12]

Recently a mouse line has been developed, which has been proposed as a model for BTHS. However this relies on shRNA knockdown of the Taz gene (TAZKD; [13, 14]). There are a number of drawbacks associated with such an shRNA knockout, particularly when studying phenotypes relevant to BTHS. The TAZKD needs to be induced and is dependent on the continued administration of doxycycline (dox) to reduce expression of the gene. However, continual administration of dox to mice has been shown to adversely affect both cardiac and neutrophil function [15]. This interferes with the study of both cardiomyopathy and neutropenia in the mouse model, which are the important clinical consequences of BTHS [13, 14, 16–18]. Further, the knockdown is incomplete as it abolishes only 89% of TAZ mRNA in cardiac and skeletal muscle meaning the allele is a hypomorph rather than a complete null [13, 16]. Perhaps most importantly, shRNA technology is prone to off-target effects, which can complicate analysis of the phenotype [19–21]. Taking all these drawbacks into account it is difficult to definitively attribute the phenotypes observed in the animals to the reduction in the levels of the Taz protein.

Studies in other animal models have shown phenotypes with some similar aspects to BTHS notably including abnormal mitochondrial morphology motor deficits and sterility in Drosophila [22, 23]. Knockdown of Taz in zebrafish produced a developmental lethal phenotype with impaired cardiac, tail and eye development [24]. Although there are similarities both these systems lack the full range of clinical symptoms seen in BTHS and generation of an appropriate mouse model should allow the function of Taz to be more closely examined in a more appropriate context.

Mitochondria play an important role during spermatogenesis and sperm function as they are the main source of energy to allow sperm motility. To allow such a burst of energy to be generated at the appropriate time, mitochondria are compacted alongside the tail of the sperm [25]. Mitochondrial compaction into the sperm tail occurs in differentiating spermatocytes following meiosis. Enzymes which regulate mitochondrial lipids have been shown to play an important role in these dynamic mitochondrial processes. For example mutation of the phosphatidic acid-preferring phospholipase A_1_, Ddhd1, causes defects in mitochondrial organisation and as a consequence spermiogenesis, the process of differentiation of haploid round spermatids into mature motile spermatozoa ([26]).

Meiosis is an essential stage of germ cell formation when genetic material is exchanged between sister chromatids to mix the gene pool of the next generation. During this process there is also extensive reprogramming of DNA methylation of imprinted genes. These epigenetic modifications leave cells vulnerable to the reactivation of endogenous mobile genetic elements such as retrotransposons, and consequent DNA damage. Keeping the integrity of the genome for the following generation is crucial during germ cell differentiation. To avoid compromising the integrity of the genetic material meiotic cells have developed a mechanism based on PIWI-like proteins and piRNAs (Piwi-interacting RNAs) which specifically recgonises and degrades transposon mRNA. Mouse orthologs of the Piwi family are Miwi (Mouse piwi, also known as Piwil1), Mili (Miwi-like, also known as Piwil2) and Miwi2 (also known as Piwil4). Deficiency in any of those proteins leads to male sterility [27], spermatocyte differentiation arrest [28] and the compromising of the genome due to overexpression of transposable elements [29]. Furthermore this process has also been shown to be dependent on the activity of MitoPLD, a mitochondrial enzyme which has been shown to act as a cardiolipin hydrolase ([30]).

BTHS is a complex multisystemic disease. We designed a mouse knockout to allow the study of Taz function in the context of appropriate tissues. Injection of targeted ES cells into blastocysts resulted in the production of high percentage chimeras. However the animals did not transmit the targeted allele through the germline.

Here we demonstrate that the high percentage chimeras were sterile as a result of a deficit in germ cell differentiation. Furthermore Taz deficient ES cells have a deficit in male germ cell differentiation in *vitro*. Germ cell differentiation is blocked at the onset of meiosis and associated with high levels of DNA damage.

## Materials and Methods

### Ethics Statement

All experiments were conducted according to the Animal (Scientific Procedures) Act 1986, under Project License 40/4293 reviewed and approved by the University of Glasgow Ethical Review Process, and the UK Animal Procedures Committee (London, UK). The UK Home Office code of practice for the housing and care of animals used in scientific procedures was followed. We adhere to the ARRIVE guidelines for reporting animal research. Animals were housed in IVC cages with temperature-controlled conditions under a 12-h light/dark cycle with free access to drinking water and food. Where appropriate, for example during a surgical procedure, isoflourane inhalational anaesthesia was administered along with a post operative analgesic, carprofen. At the end of procedures animals were euthanised according to Schedule 1 of the Animal (Scientific Procedures) Act 1986. All proceedures were continually reviewed to ensure best practice was followed. We adhere to the ARRIVE guidelines for reporting animal research.

### Taz knockout construct and ES cell details and transfection

To generate a targeting vector for conditional knockout of Taz, we created a universal vector (pFlex-DTA) by modification of pFlexible [31]. We firstly replaced the FRT-PURO *Δ*TK-FRT cassette with a Neo cassette (from pL452, [32]) then introduced a DTA (negative selection) cassette from pROSA26.1 [33] into the backbone of the plasmid. Three homology arms for Taz (5’, conditional and 3’) were generated by recombineering in DY380 *E*.*coli* [32] to retrieve the sequences from BACs containing the murine TAZ gene. These arms were then serially cloned into pFlex-DTA.

The linearised Taz targeting vector was electroporated into HM1 ESC [34] and colonies selected under G418. Surviving colonies were screened for successful targeting by PCR across the 5’ and 3’ ends of the construct and across the site of the isolated (5’) loxP site. For 5’ and 3’ screening, the internal oligos annealed to sequences within the Neo cassette and paired with genomic DNA sequences outwith the homology arms.

### HRPT-DAZL construct, ES transfection and clone selection

To allow controlled expression of Dazl, a targeting construct was generated placing the Dazl cDNA downstream of a CAG promoter ans a lox-STOP-lox cassette [35]. This construct was electroporated into Taz^Neo^ ES cells and colonies selected under HAT medium. Surviving colonies were screened for successful targeting by PCR at both the 5’ and 3’ sides. Dazl was expressed following Cre deletion of the stop cassette following electroporation of Taz^Neo^; Hprt-Dazl cells with circular pCAGGS-Cre-IRES-puro (gift of Prof. A. Francis Stewart, Technische Universitaet Dresden). Deletion of the stop cassette was confirmed by PCR across the position of the cassette.

### Primary antibodies

Rabbit polyclonal anti-Dazl (ab34139) antibody was purchased from Abcam Plc, Cambridge, UK. Rabbit polyclonal anti-Hook1 (HPA018537) antibody, mouse monoclonal anti-*β*-tubulin isoform III (T5076), polyclonal anti-Actl7B (HPA021803) were purchased from Sigma-Aldrich, UK. Rabbit polyclonal IgG anti-Pgk2 (F-25) (sc-133905), rabbit polyclonal anti-*γ*H2ax (ser139) (sc-101696), rabbit polyclonal anti-Hprt (FL-218) (sc-20975) and goat polyclonal anti-3-*β*-HSD (P-18)(sc-30820) were purchased from Santa Cruz Ltd. Rabbit monoclonal anti-*β*-actin (13E5) was purchased from Cell Signalling Technology, Beverly, MA. Monoclonal mouse anti-human Wilm’s Tumour 1 (WT 1) protein clone 6F-H2 was purchased from Dako, Ely, UK. Monoclonal mouse anti human Acrosin (AMC-ACRO-C5F10-AS) was purchased from biosonda, Santiago, Chile. Anti-Sycp-3 (NB300-232) was purchased from Novus Biologicals Ltd, Cambridge, UK. Rabbit anti-Orf-1 Line1 antibody was a kind present from Alex Bortvin, Carnegie Institution of Washington, Department of Embryology, Baltimore, MD 21218, USA. All antibodies were used to a 1/10000 dilution for Western blotting. All antibodies were used to a 1/400 dilution for immunocytochemistry and immunochemostry.

### Secondary antibodies

For immunocytochemistry the antibodies were commercially sourced from Jackson sLaboratories Ltd: FITC conjugated affiniPure Donkey anti-rabbit IgG (H+L) code 711-095-152, TRITC conjugated affiniPure Donkey anti Goat IgG (H+L) code 705-025-147, TRITC conjugated affiniPure anti-rabbit IgG (H+L) code 711-025-152 and FITC conjugated affiniPure Donkey anti mouse IgG (H+L) code 715-095-150 For western blotting: the antibodies were purchased from Amersham Ltd: ECL peroxidase labelled anti-rabbit antibody and ECL peroxidase labelled anti-mouse antibody All antibodies were used to a 1/10000 dilution. For immune-histo/cytochemistry the secondary antibodies were purchased from Dako, Ely, UK. All antibodies were used to a 1/400 dilution.

### Western Blotting, immunocytochemistry and immunohistochemistry

Western blotting, and immunocytochemistry were performed as previously described in [36].

For immunohistochemistry, the chimeras were sacrificed at specified time. Tissue was dissected and fixed in 10% neutral formalin buffer. Tissues were then embedded in wax and cut in microtome at 10*μ*m sections. Following immunochemistry was performed using Vision Cytomation Envision system from DAKO, Carpinteria, CA (ref K4006 for secondary anti mouse HRP kit and ref K4010 for secondary anti rabbit HRP kit). Briefly, the sections were dewaxed in consecutive baths of xylene and decreased amount of ethanol, followed by a rinse in water. Haptenes were exposed in citric solution (PT module buffer 1 citrate buffer, ThermoScientific, Paisley, UK) in pressure cooker for 10 min. unspecific binding was avoided by incubation with 0.03% hydrogen peroxide. The sections were washed successively in TBST and blocked with 5% goat serum for 30 min. After 2 washes with TBST the sections were incubated overnight with primary antibodies at 1/400 dilution at 4C. The sections were then washed again with TBST and incubated with secondary antibody for 30min. They were washed a further twice with TBST and staining was revealed by incubation with DAB and chromogen at an equal ratio of 1 to 1. The reaction was stopped in water and the sections were counterstained with Meyers Haematoxylin. After several rinses in water the sections were dehydrated in increasing alcoholic solutions and mounted in histomount. 6 testis analysed coming from different low and high chimeras. Each staining was at least repeated twice and picture taken representative of the pattern of expression.

### Cardiolipin profiling

Taz^Neo^ and HM-1 parental ES cells were grown gelatin to confluency on T175flasks, Corning, Amsterdam, The Netherlands. Cardiolipin profiling was performed has previously described in [37].

### RT-PCR

RNA was extracted from confluent 10cm dishes cell pellet using Qiagen RNA mini kit according to the manufacturer instruction. DNA was degraded prior to retrotransciption using Dnase RNAse-free from Roche, Burgess Hill, UK. cDNA synthesis was performed using SuperscriptIII kit from Invitrogen, Paisley, UK or iScript cDNA synthesis Kit from BioRad, Hemel Hempstead, UK. RT-PCR was performed using GoTaq polymerase from Promega, Southampton, UK. Primer details are available on request.

### Microscopy

All sections and stained coverslips were observed under Olympus BX51 FL Microscope/CellF microscopes.

### P16 Nuclear Spreads

Chimera injection for P16 testis staining and nuclear spread analysis: 2 different clones were injected to produce Taz Neo chimeras. Pups were sacrificed at P16 testes dissected. Procedure was as described in [38]. Briefly, the testes were dissected and removed from the capsule. Differentiating spermatocytes were isolated by section and incubation in trypsin. Individual cells were allowed to attach to 0.1% gelatin coated coverslips prior to drying and staining. Coverslips were fixed in 100% cold methanol for 10 min and subsequently blocked with 1%BSA (fraction V, Roche) in PBS. The coverslips were incubated with 1/400 diluted SYCP-3 antibody for 45min, washed 3 times in PBS and incubated with anti-Rabbit secondary antibody diluted to 1/500 for 30 min, washed 3 times in PBS and mounted onto hard mount medium containing DAPI (Vectashield, Hard set +DAPI H-1500, Vector laboratories, Burlingame, CA,USA). All stained cells were numbered and assessed for their developmental stage using an Olympus BX51 FL Microscope.

## Results

### Generation of a Taz Knockout Stem cell line

**Fig 1:**
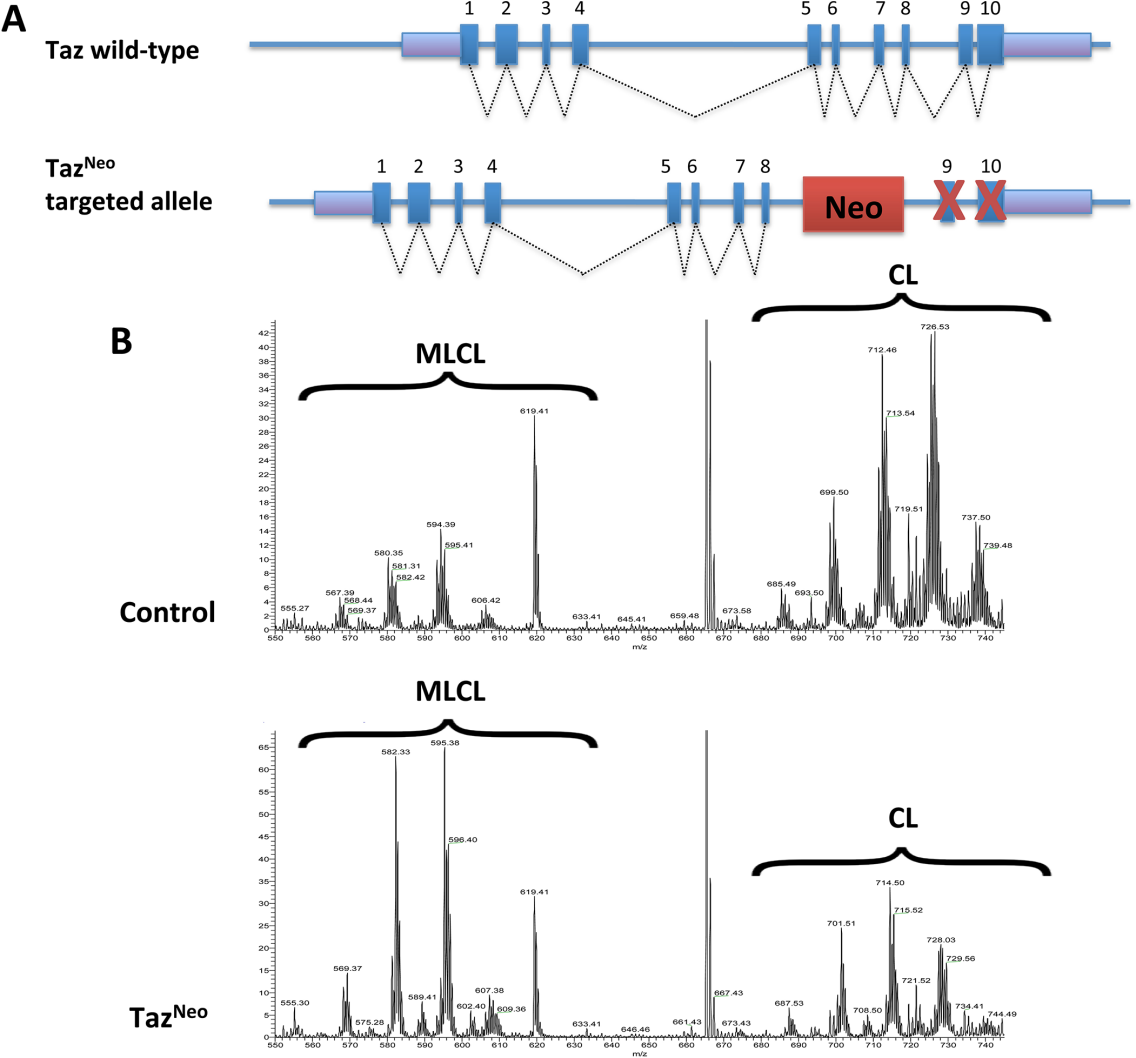
Taz^Neo^ ES cells have a reduction of cardiolipin levels. (A) Diagram of mouse taz wild-type and Taz^**Neo**^ alleles. While all 10 exons of the wild-type gene are normally expressed, the insertion of the neo cassette prevents proper expression of Taz RNA (S1 Fig). (B) Cardiolipin profile of Taz^**Neo**^ (bottom) ES cells confirms Taz deficiency by showing the accumulation of MLCL and reduction of mature CL as compared to control (top).

To allow the charatcterisation of Taz gene function we generated a knockout allele of the Taz gene (Taz^Neo^) using a targeting vector which introduces a Neo cassette between exon 8 and 9 of the mouse sequence of the X-linked Taz gene (ENSMUSG00000009995). Although originally designed as a conditional knockout we subsequently discovered that the insertion disrupts Taz function, so the allele functions as a complete knockout allele rather than a conditional. The targeting vector was linearised and introduced into HM1 embryonic stem cells (Fig 1A and S1 Fig). Appropriate targeting was confirmed by screening the ES cells at the 5’ and 3’ sides (Fig 1A and S1 Fig).

### Genetic manipulation resulted in disruption of Taz expression and male sterility

Targeted Taz^Neo^ embryonic stem cells were then injected into C57BL6/J blastocysts and chimeras were identified by coat colour (S2B Fig). Following blastocyst microinjection and embryo transfer, 29 chimeras were produced from 108 injected blastocysts (26%; compared to an average chimera production rate of 15% from injected blastocysts). Furthermore of these chimeras 22 of the 29 were considered to be high percentage and have an ES cell contribution of >70%, as judged by coat colour (S2B Fig). Although we were readily able to generate high percentage chimeras, the targeted allele did not transmit through the germline. To exclude the any possibility that these results could be attributed to a linked mutation arising the ES cells during culture, we injected a further 3 ES cell clones originating from 2 separate ES cell transfections. Taken together a total of 62 chimeras were produced and following breeding of representatives from each of these clones no transmission of the Taz^Neo^ targeted allele was observed. Lower percentage chimeras sometime gave non-germline litters but high percentage chimeras did not produce any offspring even after being in breeding for prolonged time periods, suggesting these animals were sterile.

The Taz gene has previously been shown to affect male fertility in Drosophila and as the Taz gene is on the X-chromosome, there is only a single copy of the gene in the HM1 embryonic stem cells. Taken together these data suggested that disruption of the Taz gene may underlie the sterility observed in the male chimeras. In order to test this we performed an RT-PCR (S1C Fig). Although expression of Taz is readily detectable in wild-type ES cells, no expression is detectable in ES cells with the Taz^Neo^ allele.

As Taz functions as a phospholipid–lysophospholipid transacylase, a biochemical effect of Taz deficiency, is the reduction in levels of polylysocardiolipin (CL) and the simultaneous increase in the levels of monolysocardiolipin (MLCL) species [39]. In order to assess the consequences of the Taz^Neo^ modification in the targeted ES cells, stem cell lipid extracts were subjected to LC-MS/MS to measure the relative abundance of CL and MLCL phospholipids. In the Taz^Neo^ cells there was a dramatic decrease in the CL and an increase of the MLCL levels, as well as a shift toward a more saturated form of the lipids (Fig 1B). These are similar to the changes identified in BTHS patients [39].

### Spermatocyte differentiation was compromised in Taz deficient mice

As the high percentage chimeras were infertile we analysed spermatogenesis in the Taz^Neo^ knockout mice. Histological examination demonstrated that there was a substantial reduction in the size of Taz^Neo^ testes compared to controls (S2A Fig), suggesting a deficiency in testes and sperm development. Histology of chimeric hearts showed no major difference between control and TAZ^Neo^ (S2C Fig). It should be noted however that Hprt, which can be used as a marker for the ES cells used in this study is not expressed in the heart. Consequently we are not able to say definitively whether the hearts in the chimeras originate from the introduced Taz^Neo^ stem cells or the wild-type host blastocyst. As we did not observe any major histological phenotype in the heart, and also we not able to determine accurately the origin of the heart tissue, we did not analyse the chimeric hearts in detail for this study.

**Fig 2:**
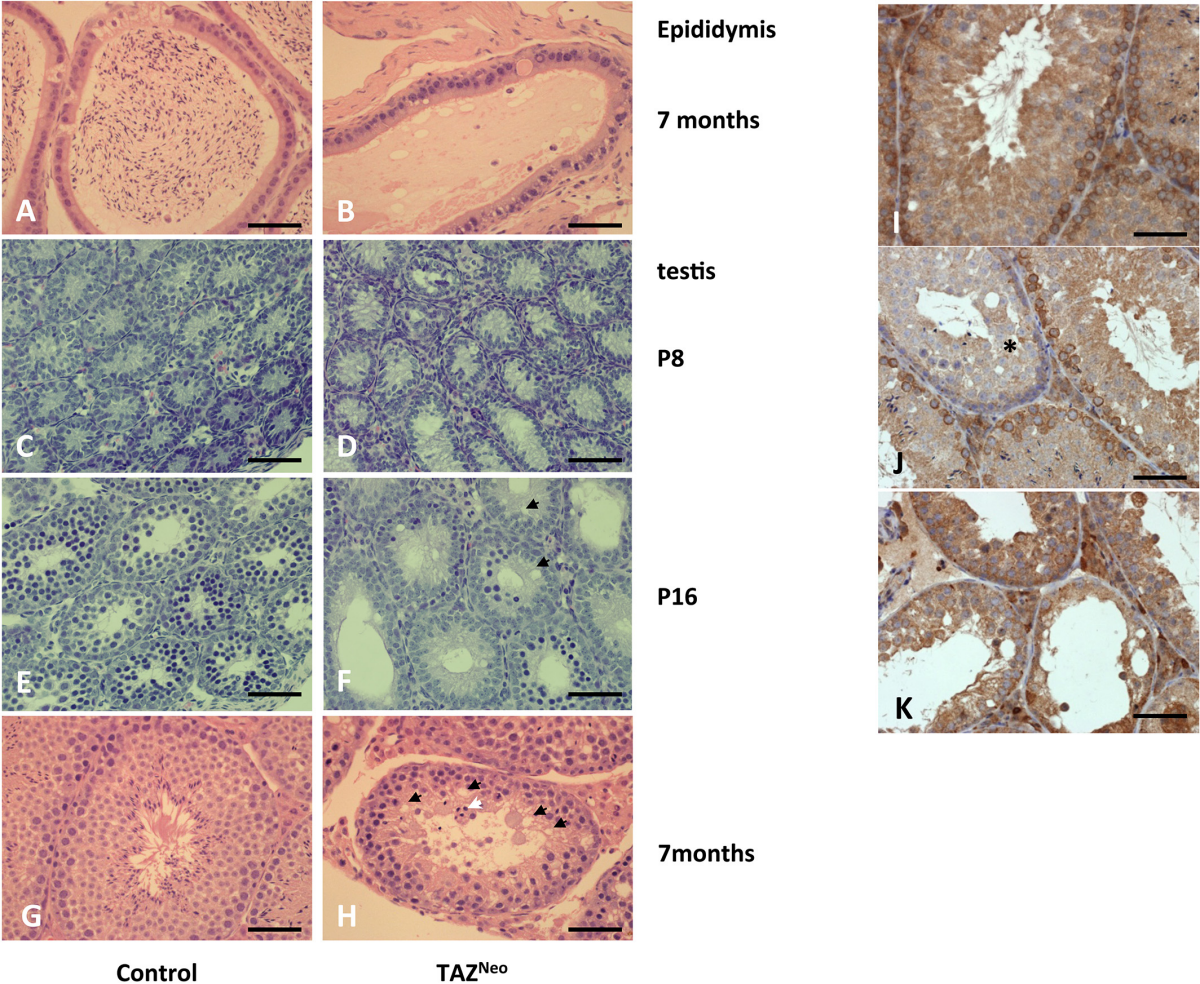
Taz deficient chimeras fail to produce mature sperm. Left panels: Histology sections of control (A,C,E,G) and Taz^**Neo**^ tissue (B,D,F,H) showing the epididymis (A,B), P8 (C,D), P16 (E,F) and adult testis tubule (G,H). Sections through the epididymis show a complete lack of differentiated sperm in the lumen of the Taz^**Neo**^ epididymis (B). White arrowhead: multinuclear cells. Black arrows: vacuoles. Scale bars: 250*μ*m. Right panels (I, J, K) show the high contribution to chimerism of Taz deficient cells in chimera testis: HPRT staining of wild type SV129P2 adult testis (I), of a low level of chimerism testis (J) (defective tubule harbouring numerous vacuoles is negative for HPRT staining*) and of a high level of chimerism testis (K). Scale bar: 250*μ*m

Hematoxylin and eosin staining of fixed sections of control and Taz^Neo^ testes revealed that the epididymis of Taz^Neo^ mice did not display any mature sperm explaining their sterility (Fig 2A and 2B). Sections through adult seminiferous tubules revealed that Taz^Neo^ tubules are less numerous than their control counterparts (Fig 2G and 2H).

Wild-type tubules display normal morphology with a layer of spermatogonia surrounding progressively differentiating cells, termed spermatocytes. Towards the lumen of the tubules, spermatids with elongated tails are visible. In contrast in the Taz^Neo^ tubules no spermatids and very few differentiated spermatocytes are visible. The layer of spermatogonia and early spermatocytes appears enlarged with cells being displaced from the basement membrane. Numerous vacuoles are visible in the luminal region where maturing spermatocytes would normally be located. (Fig 2H, *black arrows*). Large multinucleate cells are also visible adjacent to the lumen of the tubule (Fig 2H, *white arrow*). Prior to the onset of meiosis around postnatal day 8 there was no obvious difference between the developing seminiferous tubules in wild-type and Taz^Neo^ testes (Fig 2C and 2D). After the onset of meiosis however, Taz^Neo^ testes diplayed aberrant development with vacuole formation (Fig 2E and 2F).

In order to validate that the defects in germ cell differentiation observed in the chimeras are in tissue derived from the injected Taz^Neo^ stem cells, we stained the tissue with an antibody to Hprt. The HM1 embryonic stem cell line used in this study has a deletion of the Hprt locus on the X-chromosome [40]. In wild-type mice, spermatogonia and early spermatocytes express Hprt (Fig 2I), but this is subsequently switched off in the differentiated cell types as a result of X-chromosome inactivation during meiosis. All the seminiferous tubules from high percentage chimeras display differentiation defects, and the spermatogonia and spermatocytes are all negative for Hprt (Fig 2K) demonstrating that the germ cells contain the Taz^Neo^ targeted allele. In lower percentage chimeras which contain a mix of both wildtype and Taz^Neo^ cells, the tubules with defective germ cell differentiation are negative for Hprt, indicating that these are derived from the injected stem cells. Adjacent tubules with normal morphology are Hprt positive and therefore derived from the host wild-type cells (Fig 2J).

**Fig 3:**
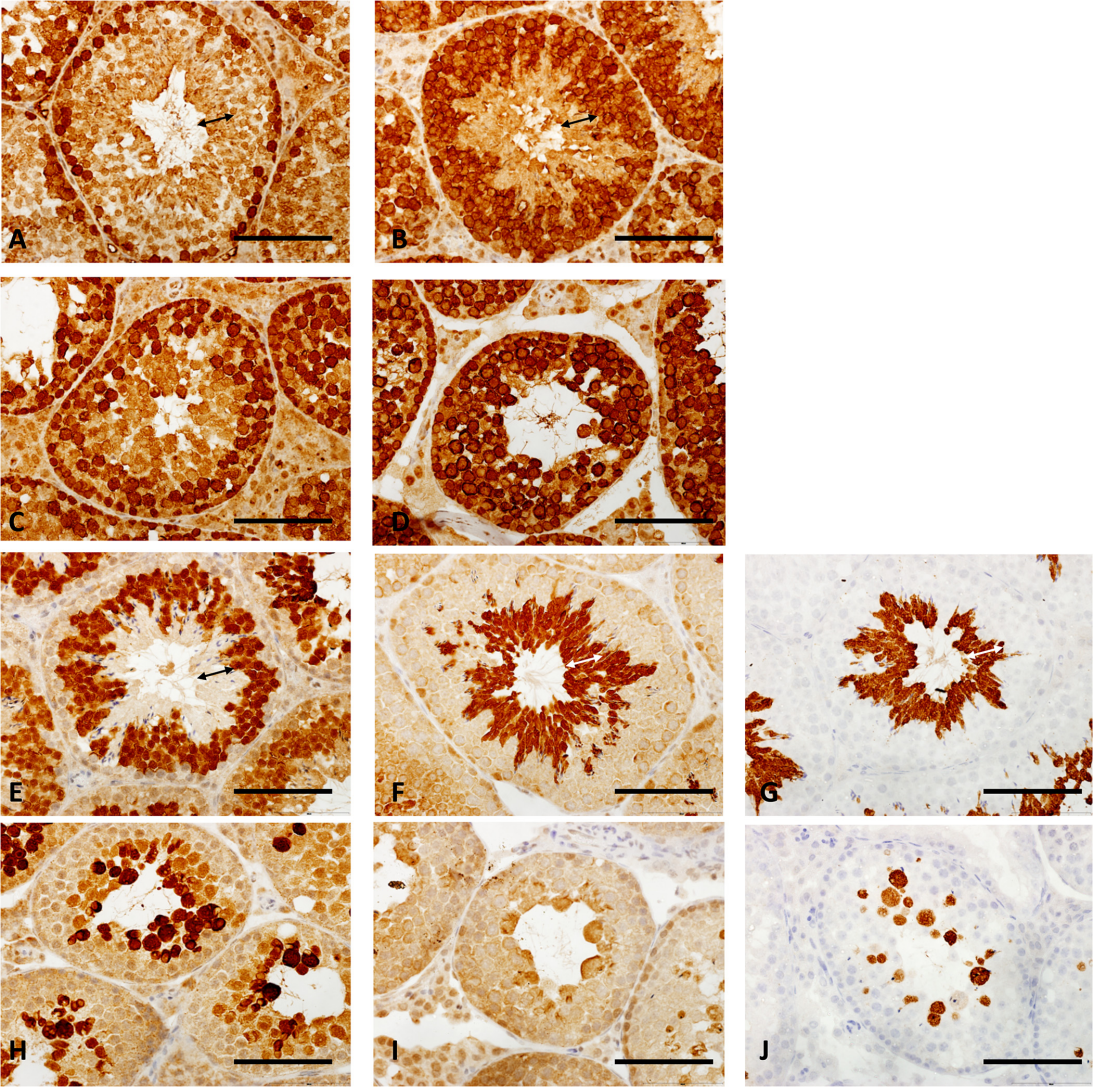
Taz^Neo^ spermatocytes do not differentiate to the round spermatid stage. Immunohistology sections of SV129P2 wild type (A,B,E,F,G) and TAZ^**Neo**^ testis (C,D,H, I and J) showing the staining of sperm germ cells marker, Dazl (A,C), Primordial germ cell marker, Vasa (B,D), mature germ cell markers Actl7B (E,H), Hook1 (F,I) and Pgk2 (G,J). Double arrows on each of the wild-type sections (A,B,E,F,G) indicate the zone of differentiating spermatozoa absent in the Taz^**Neo**^ tubules. Scale bar: 250*μ*m

A defect in proper differentiation of spermatocytes was confirmed by immunostaining the tissue sections. As the morphology of the tubules in the Taz^Neo^ mice is severely disrupted accurate staging of the seminiferous epithelial stages is not really possible. A description of the cell types which should be positive for the markers used is provided (S1 Table). Although markers expressed in spermatogonia and pre-meiotic spermatocytes are present, these are expressed in abnormal patterns in the testis tubules of Taz^Neo^ mice. The number of cells expressing Dazl is increased and the staining cells are no longer only confined to the base of the tubule as in the wild-types (Fig 3A and 3C). The zone of differentiating spermatozoa (indicated by the arrows on the corresponding wild-type sections) is consistently absent in all the Taz^Neo^ sections (Fig 3). The pattern of Mvh (mouse VASA homologue; Ddx4) in the Taz^Neo^ tubules expression is disrupted and most of the remaining cells in the tubule now express Mvh (Fig 3B and 3D). Very little staining was apparent for Actl7b, a protein expressed in haploid round spermatids in the Taz^Neo^ testis section (Fig 3E and 3H). There is also no staining apparent for the differentiated sperm markers Hook1 (Fig 3F and 3I) and Pgk2 (Fig 3G and 3J), except a small amount of staining in cells with aberrant morphology, frequently large multinucleate cells. Taken together these data support the conclusion that the Taz^Neo^ germ cells are not able to differentiate fully, and are not able to transit through meiosis to form haploid spermatids.

**Fig 4:**
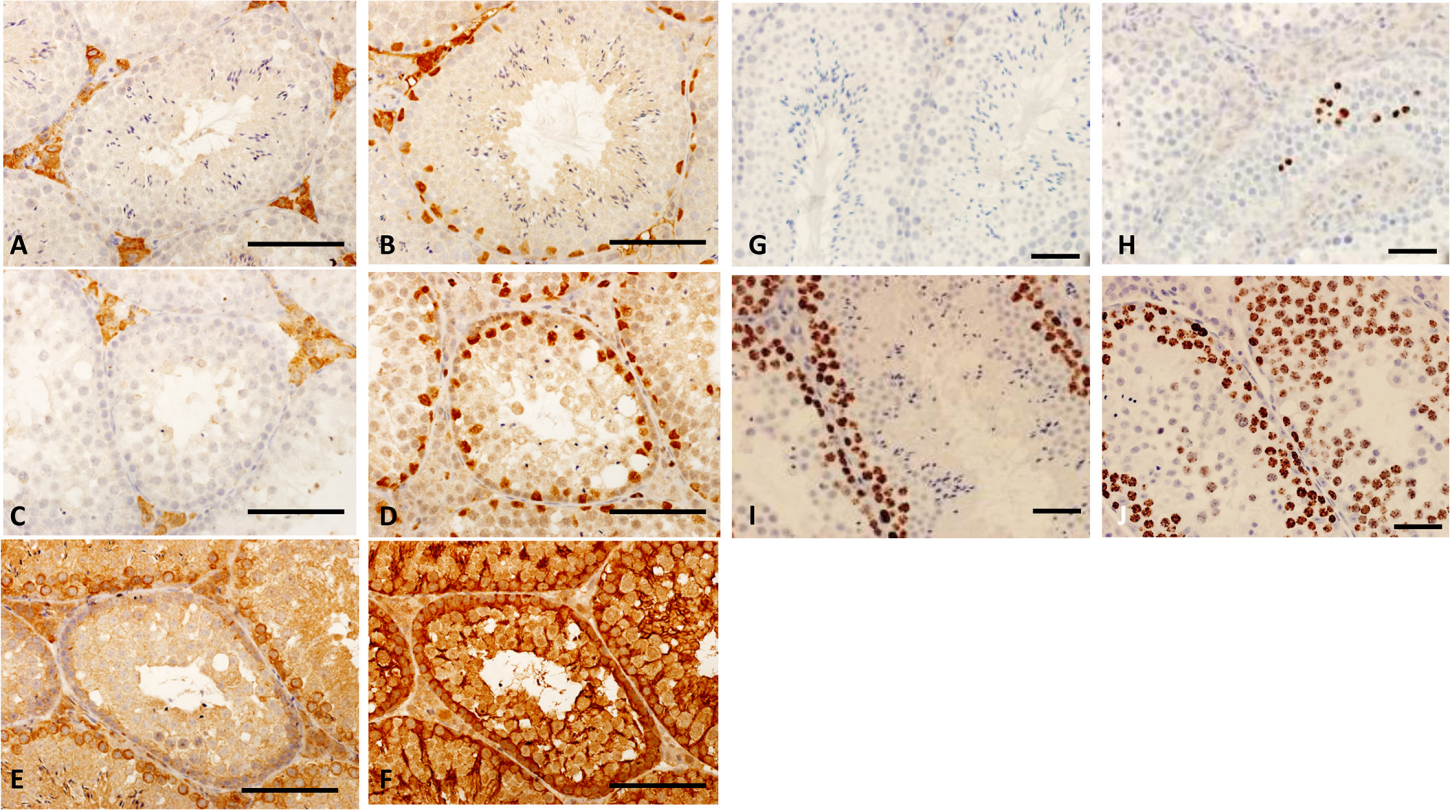
Leydig and Sertoli cells are not affected by Taz deficiency. Immunohistology sections of SV129P2 wild type (A,B, G,I) and Taz^**Neo**^ testis tubules (B,D, H and J) showing the staining of Leydig cell marker 3*β*-HSD (Hsd3b6) (A,D), sertoli cell nucleus Wt1 (B,D). Recipient Sertoli cell non-contribution in Taz^**Neo**^ testis tubules (E, F): low chimera section showing wild type and defective tubules as stained with Hprt (E) and adjacent section stained for Tubb3 showing sertoli cell cytoplasm (F). Taz^**Neo**^ semiferous tubules showed increased cell death. Caspase-3 staining of sections of SV129P2 wild type (G) and Taz^**Neo**^ (H) showing the occurrence of cell death (brown nuclear staining) in Taz^**Neo**^ seminiferous tubules. Ki67 immunostaining of the control tubule is found restricted to dividing spermatogonia in the basal region of the tubules (I) as Taz^**Neo**^ tubules display positive staining through the tubule (J). Scale bar: 250*μ*m.

In contrast to the effect of the Taz^Neo^ allele on germ cell development, accessory cells in the testis appear normal. Leydig cells (Fig 4A and 4C) and Sertoli cells (Fig 4B and 4D) were not significantly affected in the sections of Taz^Neo^ tubules as seen in staining for Wt1 and Hsd3b6 (3*β*-HSD), respectively. Furthermore Sertoli cells in mutant tubules in low percentage chimeras appear normal, as judged by Tubb3 staining, which identifies Sertoli cell cytoplasm (Fig 4E and 4F). Taz^Neo^ germ cells are properly surrounded by Sertoli cell cytoplasm. Furthermore all the tubules, even in low percentage chimeras have mutant germ cells. Taken together these data suggest that the differentiation defect observed in the mice is as a result of a defect in the germ cells themselves rather than a defect in accessory cells of the testis.

To determine if an increase in cell death was responsible for the lack of differentiated cell types observed in the Taz^Neo^ tubules, we examined the levels of apoptosis. Cleaved caspase 3 staining demonstrated an increase in apoptotic cell death often adjacent to the vacuoles in Taz^Neo^ seminiferous tubules (Fig 4G and 4H). In the testis the proliferation marker Ki67 is present is in spermatogonia and all stages throughout meiosis, subsequently being turned off in round spermatids as the cells start to differentiate. In the Taz^Neo^ tubules there is an expansion of the zone of expression and number of cells expressing Ki67, suggesting the cells fail to transit through meiosis properly (Fig 4I and 4J).

### *In vitro* differentiation of Taz deficient ES cells was unable to give rise to mature germ cells

In order to determine whether the defect in differentiation was intrinsic to the mutant germ cells, we directly differentiated the Taz^Neo^ ES cells into germ cells *in vitro*. To achieve this efficiently we over expressed the critical germ cell regulator, Dazl. The role of members of the DAZ family (Deleted-in-Azoospermia) is crucial in male sterility [41, 42]. These are RNA binding proteins able to modulate meiotic events and sperm differentiation. Recent studies have even shown that DAZL (DAZ-like) RNA binding protein alone is able to drive differentiation of embryonic stem cells towards the primordial germ cell lineage [43].

A lox stop lox Dazl cDNA was introduced by homologous recombination into the Hprt locus of both wild-type and Taz^Neo^ cells (S3 Fig). Expression of Dazl was activated by transfection of Cre (S3B and S3C Fig). Prior to differentiation no effect of Dazl expression was apparent on either the wild-type or Taz^Neo^ embryonic stem cells. Cells were induced to differentiate by withdrawal of the cytokine LIF from the growth medium.

**Fig 5:**
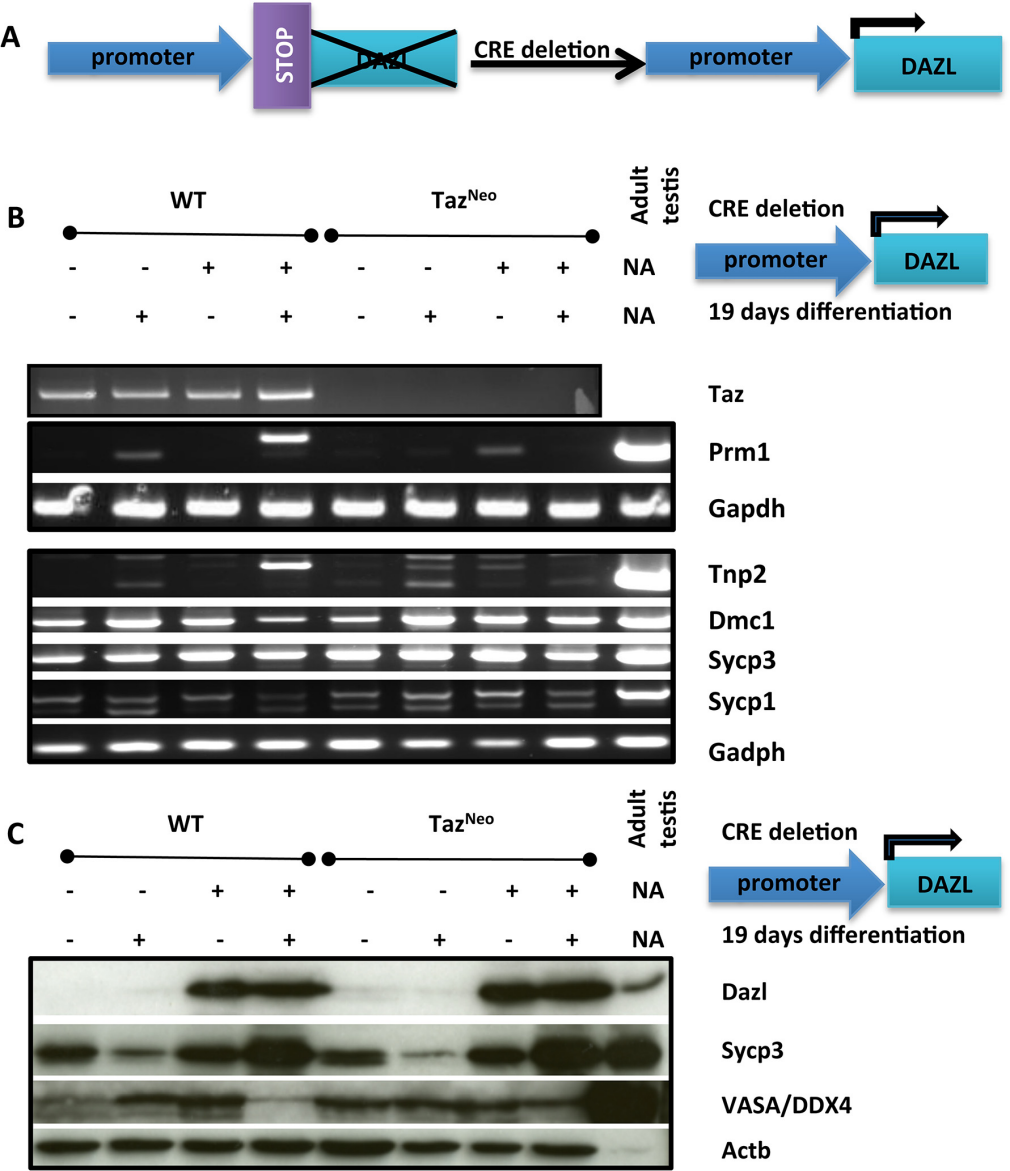
Taz^Neo^ ES cells don’t express spermiogenesis markers when differentiated in vitro. A: Top Panel: Scheme of the introduction of the flox stop Dazl construct at the HPRT locus before and after Cre deletion and recombination at the lox P sites (black arrow head). B: RT-PCR of Taz and various sperm differentiation markers (Prm1, Tnp2, Dmc-1, Sycp-3, Sycp-1) with cDNA extracted from HM1 parental ES clones or Taz^**Neo**^ ES cells before or after 19 days differentiation. C: Western blotting of wild type or Taz deficient ES cell clone protein extract with or without 19 days differentiation for Dazl, Sycp-3 or Vasa. *β*-actin is used to show equal loading.

After 19 days in differentiation medium, cells were examined for the appearance of differentiated spermiogenic markers. In the wild-type cells prior to differentiation, expression of Dazl doesn’t significantly affect the level of RNA and protein for some of the early meiotic markers, Dmc1, Sycp1 and Sycp3 (Fig 5). Upon differentiation though, protein levels decrease for these markers as a result of Dazl expression, suggesting that Dazl is promoting germ cell differentiation and transit through meiosis. When the Dazl expressing wild-type cells are differentiated the expression of spermiogenesis markers Tnp2 and Prm1 are induced. In contrast when Dazl is expressed in the differentiating Taz^Neo^ cells, although the meiotic markers are induced in a similar pattern to the wild-type cells, there is no expression of the spermiogenesis markers Tnp2 and Prm1. The differentiated sperm marker Acrosin is also induced in the differentiated wild-type ES cells, but not the Taz^Neo^ cells (S4 Fig). Taken together these data again suggest that the Taz^Neo^ cells are unable to complete meiosis, even when differentiated *in vitro*.

### Chromosome synapsis in meiosis is disrupted in Taz^Neo^ spermatocytes

**Fig 6:**
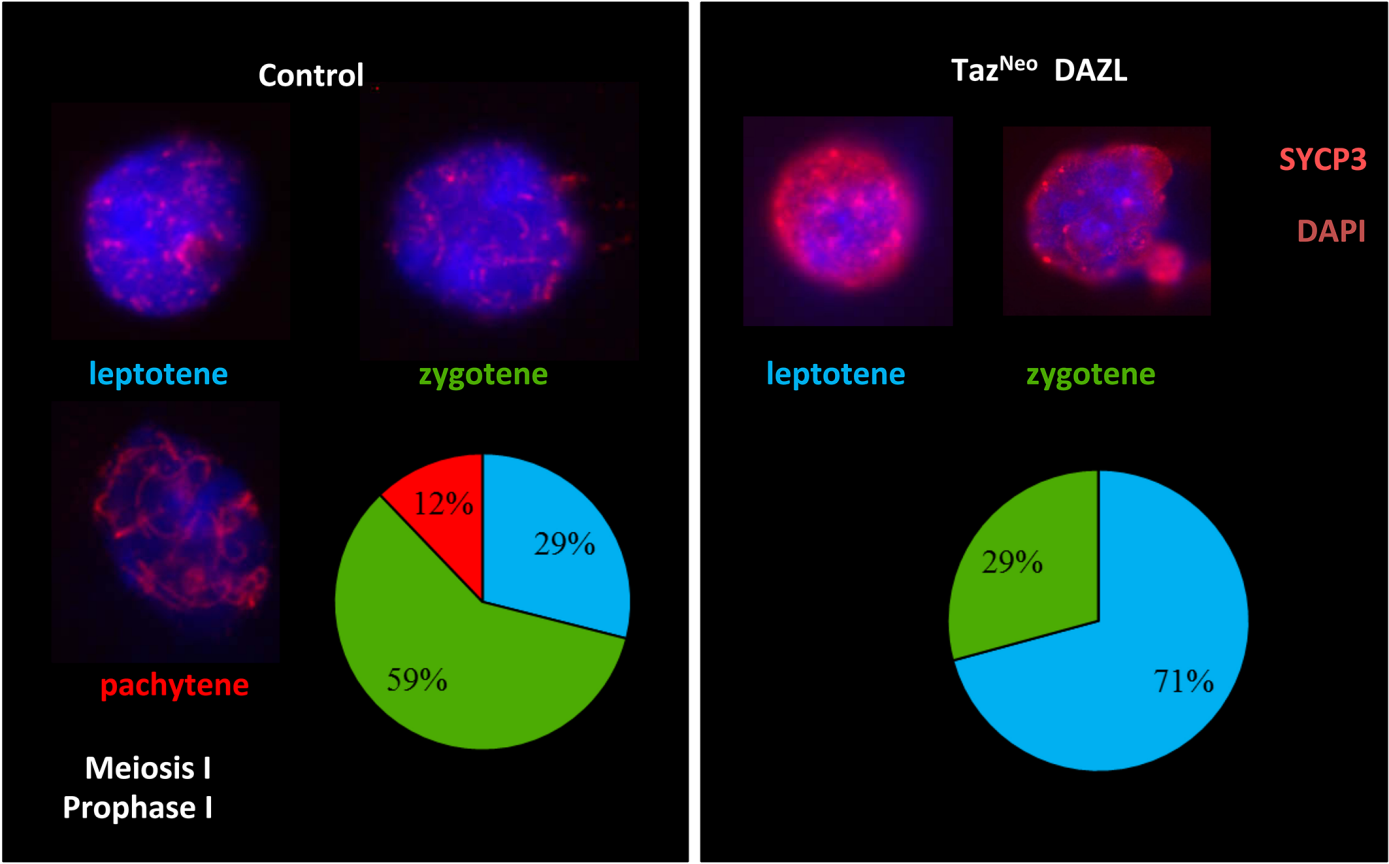
Taz^Neo^ germ cell differentiation fails before reaching the Pachytene stage of meiosis I. Sycp-3 immunostaining of nuclear spread from control (left) and Taz deficient P16 spermatocytes assessing the different stages of the Meiosis I prophase I during spermatocytes differentiation (leptotene, zygotene, pachytene). Scale bars: 50*μ*m. Proportion of cells in various prophase I stages in control or Taz deficient spermatocytes (lower right panels).

To establish more precisely what stage of spermatocyte differentiation was affected in Taz^Neo^ mice, we performed a nuclear spread of meiotic P16 spermatocytes. To assess the progress of synapsis in those cells we stained the nuclei with Sycp3, a component of the synaptonemal complex present both prior to chromosome synapsis and subsequently. In contrast to the wildtype controls where leptotene (29%), zygotene (59%) and pachytene (12%) stage nuclei were readily identifiable, the Taz^Neo^ spermatocytes were either in leptotene (71%) or zygotene (29%) stage of the first meiotic division (Fig 6). No pachytene stage meiotic spreads were observed in spermatocytes taken from the Taz^Neo^ deficient testis. We could therefore assume that Taz deficient spermatocytes never reached the pachytene stage of meiosis I. This analysis was performed twice on chimeras derived from different ES cell clones suggesting that disruption of Taz results in a consistent defect in meiosis.

### Taz^Neo^ Spermatocytes have increased levels of DNA damage and endogenous retrotransposon expression

As a block of progression through meiosis is often an indicator of increased levels of DNA damage, we were interested to investigate if the genetic integrity was compromised in Taz^Neo^ spermatocytes.

**Fig 7:**
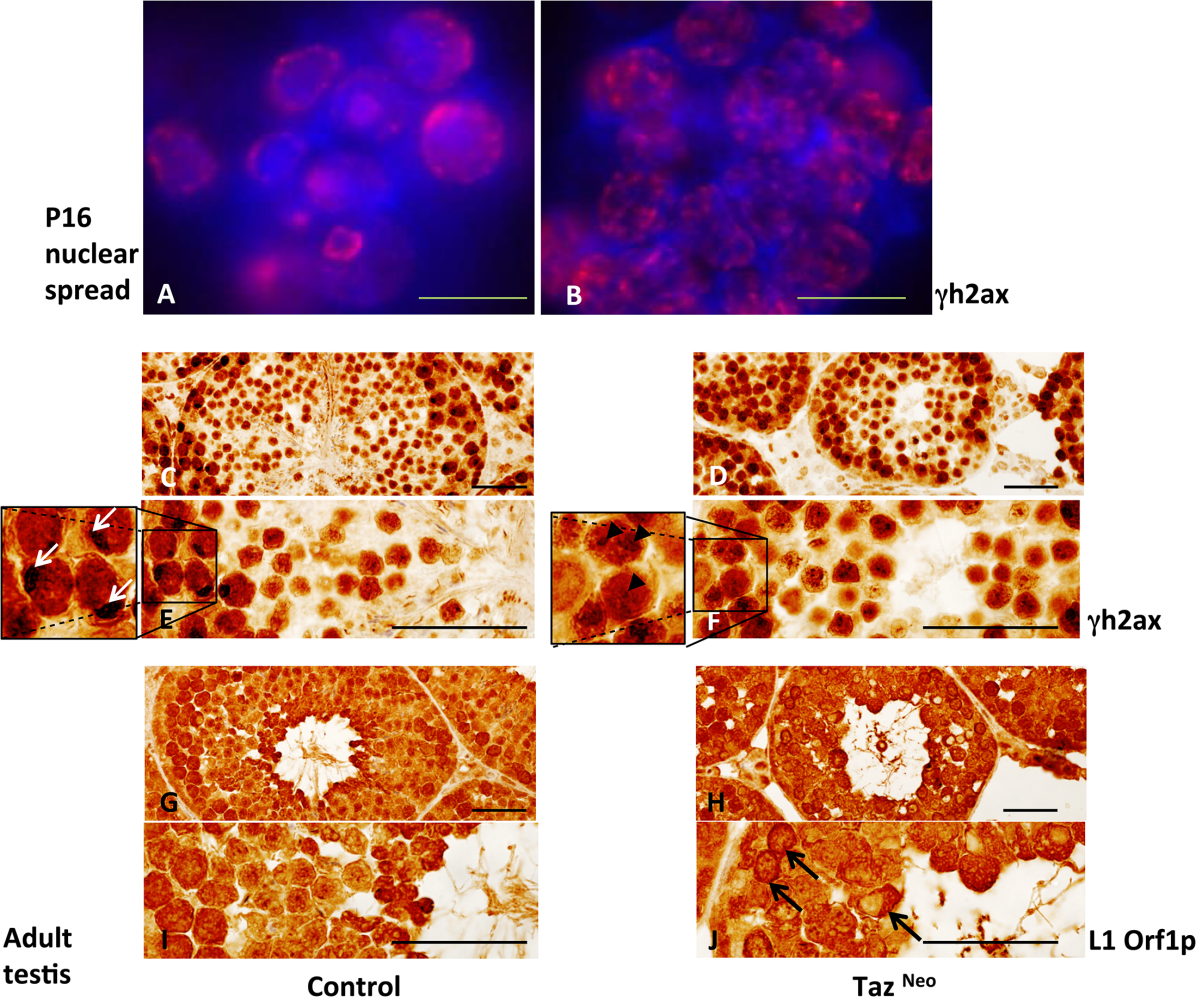
Taz deficient seminiferous tubules showed increased DNA damage due to higher L1 retrotransposon activity. A, B: immunostaining of P16 nuclear spread from control (A) or Taz^**Neo**^ testis (B) using anti-*γ*H2ax (red) antibody. Dapi nuclear stain (blue). Scale bar 50*μ*m. C-J: Immunostaining of adult testis sections of control (C,E,G,I) and Taz^**Neo**^ testis (D,F,H,J) with anti-*γ*H2ax (C-F) or anti-Orf1-Line1 (G-J) antibody. White arrows: sex bodies seen on germ cells reaching Pachytene stage (E). Black arrow heads indicating typical punctuating staining of DNA damage (F). Black arrows: Orf1 Line1 nuclear staining (J). Scale bars: 250*μ*m.

*γ*H2afx (*γ*H2ax), a histone recruited to the sites of DNA double stranded breaks, was used as a marker for the occurrence of DNA damage. Staining of P16 spermatocyte nuclei demonstrated a much greater number of *γ*H2ax foci were present in Taz^Neo^ spermatocytes compared to wild-type controls (Fig 7A and 7B). Immunostaining of tissue sections from adult Taz^Neo^ testis revealed a prolonged activation of *γ*H2ax. Wild-type testis showed highest levels of *γ*H2ax staining in the dividing spermatogonia and lower levels in the differentiating spermatocytes with sex body, formed from the inactivated X and Y chromosomes, staining most obviously in the spermatocytes (Fig 7C–7F). In contrast in the Taz^Neo^ seminiferous tubules the majority of cells stained strongly, supporting the hypothesis that meiotic failure may be as a result of increased DNA damage. The sex body typically formed during the pachytene stage of meiosis by inactivation of the unpaired X and Y chromosomes is not visible in the Taz^Neo^ sections. This again supports the hypothesis that the germ cells in the Taz^Neo^ germ cells are failing to go through meiosis properly. This is similar to the phenotype observed in the MIWI2^-/-^ animals ([29]).

DNA damage during spermatogenesis can be caused by the activity of endogenous retrotransposons. To examine this we used an antibody to Orf1p of the LINE-1 (L1). L1 retrotransposons are the most frequently occurring transposable elements in the mammalian genome and Orf1p is one of two proteins produced by L1, critical for its retrotransposition activity. The Taz^Neo^ seminiferous tubules had higher levels of Orf1p protein than wild-type and this was often localised in the nucleus of the spermatocytes in the Taz^Neo^ tubules (Fig 7G–7J). Taken together these data suggest that an increase in L1 activity is causing increased DNA damage levels resulting in meiotic failure in the Taz^Neo^ spermatocytes.

## Discussion

BTHS in humans has been attributed to the deficiency of the Taz gene function [5, 44]. Although there is variable penetrance, the syndrome is characterised by cardiomyopathy, neutropenia, skeletal myopathy and growth delay. The product of the Taz gene is a mitochondrial phospholipid transacylase. Abnormal mitochondrial lipid content and morphology are diagnostic features of the disease, and have been assumed to be the underlying cause of the clinical features. Despite this, the molecular mechanism linking the mitochondrondrial abnormalities with the characteristic features of BTHS such as cardiomyopathy and neutropenia remain largely unclear.

In order to better understand the function of Taz in the mouse, we have generated a knockout allele of the Taz gene. Chimeras generated by injection of targeted Taz^Neo^ ES cells were sterile and unable to transmit the targeted allele through the germline. Sterility in the mutant mice occurs as a result of meiotic failure of spermatocytes. The spermatocytes have increased DNA damage and increased levels of endogenous L1 activity.

The phenotype in the mouse was replicated *in vitro*, by directly differentiating ES cells to germ cells through expression of Dazl. This confirms that the deficits seen in the mutants are a direct effect of the mutation in the spermatocytes themselves rather than an indirect effect on the accessory cells in the testis. This is also confirmed by the data showing that the Leydig and Sertoli accessory cells in the testis apparently develop normally in the Taz^Neo^ seminiferous tubules.

The data presented above are all consistent with a disruption of meiosis in the Taz mutants. The onset of the phenotype in the mouse, first visible at day 16 correlates with the onset of the first wave of meiosis in the testis. Immunohistochemical characterisation of differentiation places the defect prior to the onset of spermiogenesis. This correlates with the data from the mutant ES cells, which do not express spermiogenesis markers when differentiated *in vitro*. In addition Ki67 staining suggests that mutant spermatocytes fail to exit meiosis properly.

Knockouts of a number of genes, which result in a deficit in male germ cell meiosis, have shown to be involved the synthesis of PIWI-interacting RNAs (piRNAs). piRNAs are a class of small RNAs which play a role in both post-transcriptional gene silencing and epigenetic suppression of retrotransposons, such as L1. Knockout of enzymes such as Piwil2, Piwil4 and Mael have all been shown to result in meiotic arrest, increased DNA damage and a lack of retrotransposon repression [27, 28, 45–48]. Consequently it is possible that a defect in the biosynthesis of piRNAs may underlie the phenotype observed in the Taz^Neo^ mice.

Despite this, it should be noted that there is a small amount of residual staining for the markers Hook1 and Pgk2, although the cells appear abnormal. This could potentially indicate that the TazNeo mice do not display a complete meiotic block frequently seen with knockouts of genes involved in the piRNA pathway. It should also be noted that this could also be influenced by the 129 genetic background of the ES cells which were used for this study. For example Mael^-/-^ males show a complete meiotic block in the C57BL6/J background, but on a 129 background Mael^-/-^ males show defects in spermatogenesis at later stages ([49]).

Recently Pld6 (MitoPLD), a mitochondria-localised member of the phospholipase D family, has been shown to play a role in male fertility. Pld6 mutant mice exhibit a very similar phenotype to that of the Taz mutation, including meiotic arrest of spermatocytes and increases in DNA damage and retrotransposon levels [30, 38]. Although MitoPLD has previously been shown to hydrolyse cardiolipin to the signaling lipid phosphatidic acid, more recently it has been shown to act directly as an endoribonuclease in the biosynthesis of piRNAs [50–52]. Consequently one possible explanation for the Taz phenotype is that it acts directly on the localisation or function of Pld6. Taz may also act indirectly on Pld6 by affecting substrate availability or by affecting mitochondrial morphology or function. A more in depth study of mitochondrial morphology by EM may help to clarify these possibilities.

Despite our observation that mutation of Taz results in sterility in mice, it has not been reported as a clinical feature of BTHS in humans, and some BTHS patients have had children. However the clinical features of the disease in humans are very variable and may be affected by differing genetic background in different individuals. Furthermore although the role of piRNAs in meiosis in a variety of model systems has been relatively well described there is little known about the diversity of piRNAs, their target specificity and function so it’s possible that there could be some functional differences between mouse and human [53]. In other models systems for example, it has been shown previously that mutation of Taz in the fruit fly, also results in a male sterile phenotype suggesting that there is an evolutionary conservation of function in spermatogenesis [22, 23].

Recently a transgenic mouse line expressing an shRNA for Taz has been described. This mouse line shows some similarities to the clinical signs of BTHS including a reduction in cardiolipin levels [13, 14], signs of exercise intolerance [18] and a variable cardiac phenotype [16] dependent of the dose and timing of dox used to induce expression of the shRNA. At the most severe, administration of high dox levels during embryogenesis led to perinatal lethality in a high proportion of mice. This contrasts with the results of this study using a mouse ES cell line, which expressed no RNA for Taz, and which is therefore most likely a complete null allele. We were able to generate high percentage chimeras with high efficiency, suggesting that there was no loss of chimeric animals during embryogenesis, or perinatally. Additionally, despite keeping the high percentage Taz^Neo^ mice until they were around 8 months old, these animals showed no obvious signs of illness attributable to cardiac dysfunction. Furthermore gross histological and tissue sectioning of cardiac tissue from Taz^Neo^ mice revealed no obvious gross abnormalities in the heart (S2C Fig). That said, we are not able to definitively determine that the heart tissue is completely derived from the Taz^Neo^ ES cells, so it remains possible that some or all or the hearts are generated from wild-type tissue in the chimeras.

One apparent explanation for this difference could be that the Taz shRNA has off target effects and it may be that altering the expression of other genes in addition to Taz. Consequently disruption of other genes is contributing to the cardiac phenotype observed in these animals. Of note it has previously been shown that continual administration of Dox can affect cardiac development and function, which could also be a contributory factor to this difference [15].

Another potential explanation for this difference in phenotype observed between the Taz^Neo^ targeted chimeras and the shRNA mouse model is that the shRNA knock-down would affect Taz expression in the placenta. When ES cells are injected into a host blastocyst these cells contribute to the embryo but not to the extra-embryonic tissues including the placenta [14]. This suggests that there is a possibility that expression of Taz in the placenta may be important for normal development of the embryo, and that following knockdown during embryogenesis poor placental function may even be a contributing factor in the developmental cardiomyopathy observed. There is good evidence that mitochondria are important for normal placenta function and some emerging evidence that placental function in embryogenesis is important for normal heart development [54]. Taken together these data imply that a deficit in placental mitochondria as a result of a disruption of Taz function may cause abnormal development of the heart indirectly by affecting placental function.

An alternate possibility may be the influence of the genetic background of the ES cells. Cardiomyopathy in humans is very variable in humans and the clinical severity can vary between individuals carrying the same mutation in the Taz gene. As all the experiments described here have been done in the 129P2 genetic background it is possible that this has minimised the cardiac phenotype we observed. By contrast the shRNA mouse line is maintained on the C57BL/6 genetic background [14]. If we had introduced the mutation onto a different genetic background we may see a more significant cardiac phenotype. Although it is very likely, as the chimeras analysed in this study were all high percentage, we cannot definitively rule out that there may be contribution of wild-type cells to the hearts. This could effectively have rescued any mutant phenotype. Had it been the case that the Taz^Neo^ allele resulted in a significant impairment of cardiac function, we would have expected that the high percentage chimeras would have been less likely to survive than the low percentage chimeras and we saw no evidence for this.

In the course of this study we have uncovered a new role role for Taz in male meiosis and the suppression of endogenous retrotransposons. It is possible that rather than playing a direct role in the generation of piRNAs themselves that Taz is functioning to help maintain the integrity of mitochondria which have been shown to serve as a platform for piRNA production ([30, 38, 55, 56]). Although piRNA expression and function was originally proposed to be germ cell specific, there are now a number of reports suggesting that piRNAs are expressed in other tissues [57–59]. For example piRNAs have recently been shown to play a role in memory formation in neurons [60]. Furthermore there is also evidence that inappropriate activation of retrotransposon expression in the heart may cause tissue damage [61, 62]. Taken together these data suggest the possibility that the role of Taz in suppressing retrotransposon expression, may be important in tissues other than just the germline. Consequently it is possible that a failure to suppress retrotransposons appropriately may not only be responsible for the sterility phenotype but may also contribute to the important clinical features of BTHS such as cardiomyopathy or neutropenia. Further investigation, of the roles of Taz uncovered in this study, will determine whether inappropriate expression of retrotransposons could play a role in the pathogenesis of BTHS.

## Supporting Information

S1 Fig Taz^Neo^ targeted ES cells no longer express the tafazzin gene. A: Scheme of mouse Taz^Neo^ ES cells generation and PCR screen. White box: UTR. Grey box: encoding exon. Light grey box: neomycin resistance cassette. Dark grey triangle: lox P site. Semi-circle block: FRT site. Small arrows indicate the location of PCR primers used for the ES clones screen 5’ (red) & 3’ (green). Underlying pictures show the PCR bands obtained with different clones (3B6, 3B5, 1D12 and 1B12). Control DNA is denoted as WT (wild type). B: RT-PCR of Taz^Neo^ ES cells (clone 3B5 and 3B6) confirming the loss of Taz expression. Gapdh is used to assess equal loading.
(TIF)

S2 Fig Taz^Neo^ mice have small testis and apparently normal hearts. A: Taz^Neo^ testes appear smaller than control counterparts. Scale bar: 1cm. B: Taz^Neo^ mice have a very high level of chimerism as seen by the chinchilla pelt colour and the occurrence of red eyes confirming the high contribution of HM1 ES cells in the chimeric mice. C: Heamatoxylin and eosin staining of control and Taz^Neo^ heart sections. Scale bar: 250*μ*m.
(TIF)

S3 Fig Generation of wild-type and Taz^Neo^ ES cells expressing Dazl. (A) Diagram of targeting vector used to over express Dazl in targeted ES cells (B) Western blot showing Dazl expression levels in wild-type (WT) and Taz^Neo^ targeted ES cells. Dazl (C) and Taz (D) immunostaining in WT (left panel) and Taz Neo (right panels) ES cells without (top panels) or with (bottom panels) CRE recombination.
(TIF)

S4 Fig In-vitro differentiated Taz^Neo^ ES cells don’t express the sperm terminal differentiation marker Acrosin. (A) Western blot of showing Acrosin expression in ES cells with or without Dazl expression following Cre deletion of the stop cassette and before or after 19 days of differentiation. The sample labelled +/- is a mixture of Dazl expressing and non-expressing cells. *β*-actin is used to assess equal loading. (B) Immunostaining of Acrosin (green) protein is expressed in fully differentiated Dazl (red) expressing parental HM1 ES cells (top panels) but not in Taz deficient clones (lower panels). The cells were differentiated for 19 days prior to immunostaining. Right panels show merged pictures of Acrosin (green), Dazl (red) and Dapi (blue). Scale bar: 50*μ*m.
(TIF)

S1 Table Expression of marker genes used for staining Taz^Neo^ testis tubules. (PDF)
