# Supplementary figures and images for: Mouse Tafazzin Is Required for Male Germ Cell Meiosis and Spermatogenesis
Source: PLoS One. 2015 Jun 26;10(6):e0131066. doi: 10.1371/journal.pone.0131066 (PMC4483168; doi:10.1371/journal.pone.0131066)

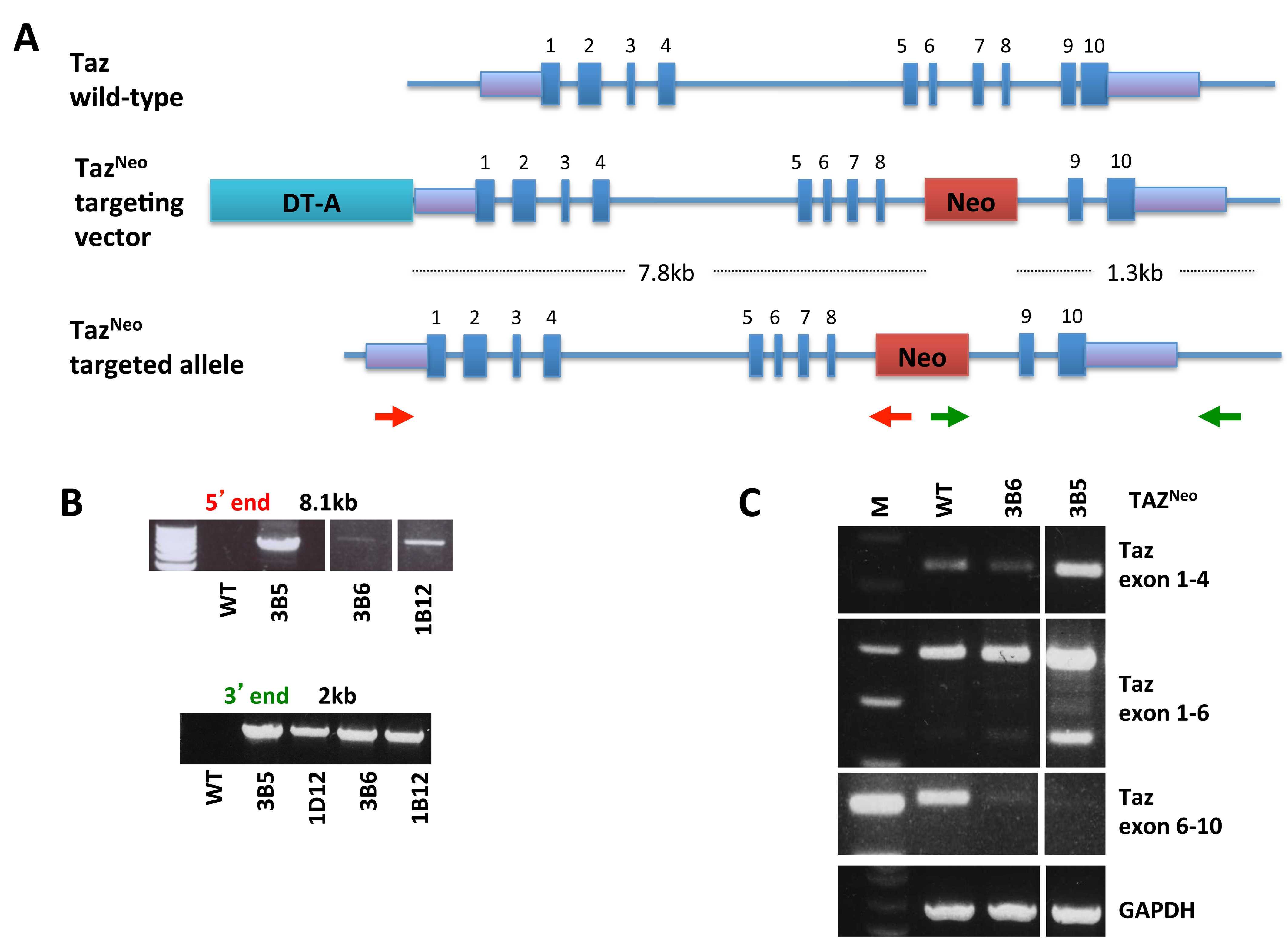

Supplement: S1 Fig — A: Scheme of mouse TazNeo ES cells generation and PCR screen. White box: UTR. Grey box: encoding exon. Light grey box: neomycin resistance cassette. Dark grey triangle: lox P site. Semi-circle block: FRT site. Small arrows indicate the location of PCR primers used for the ES clones screen 5’ (red) & 3’ (green). Underlying pictures show the PCR bands obtained with different clones (3B6, 3B5, 1D12 and 1B12). Control DNA is denoted as WT (wild type). B: RT-PCR of TazNeo ES cells (clone 3B5 and 3B6) confirming the loss of Taz expression. Gapdh is used to assess equal loading. (TIF) [file pone.0131066.s001.tif]

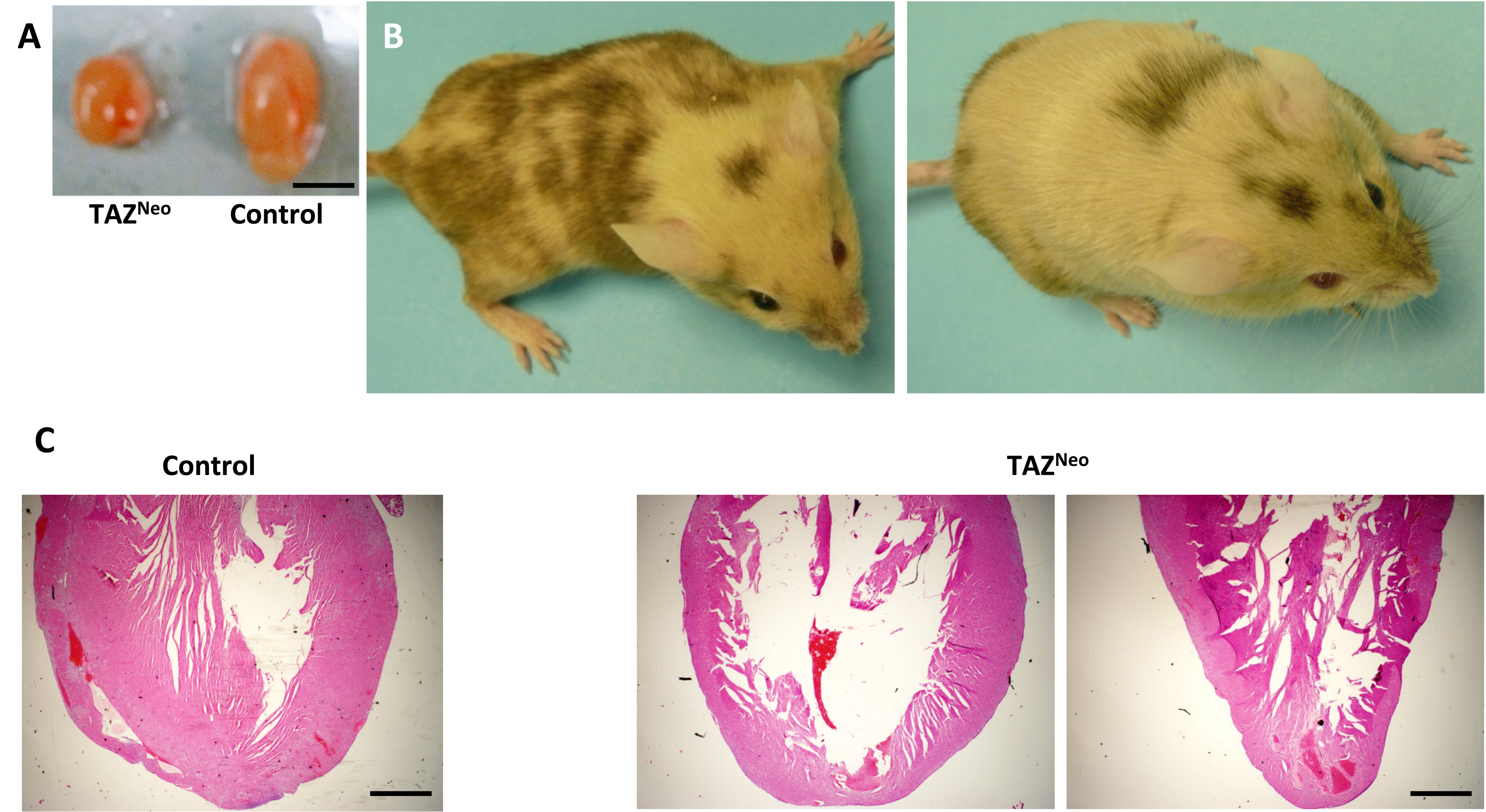

Supplement: S2 Fig — A: TazNeo testes appear smaller than control counterparts. Scale bar: 1cm. B: TazNeo mice have a very high level of chimerism as seen by the chinchilla pelt colour and the occurrence of red eyes confirming the high contribution of HM1 ES cells in the chimeric mice. C: Heamatoxylin and eosin staining of control and TazNeo heart sections. Scale bar: 250μm. (TIF) [file pone.0131066.s002.tif]

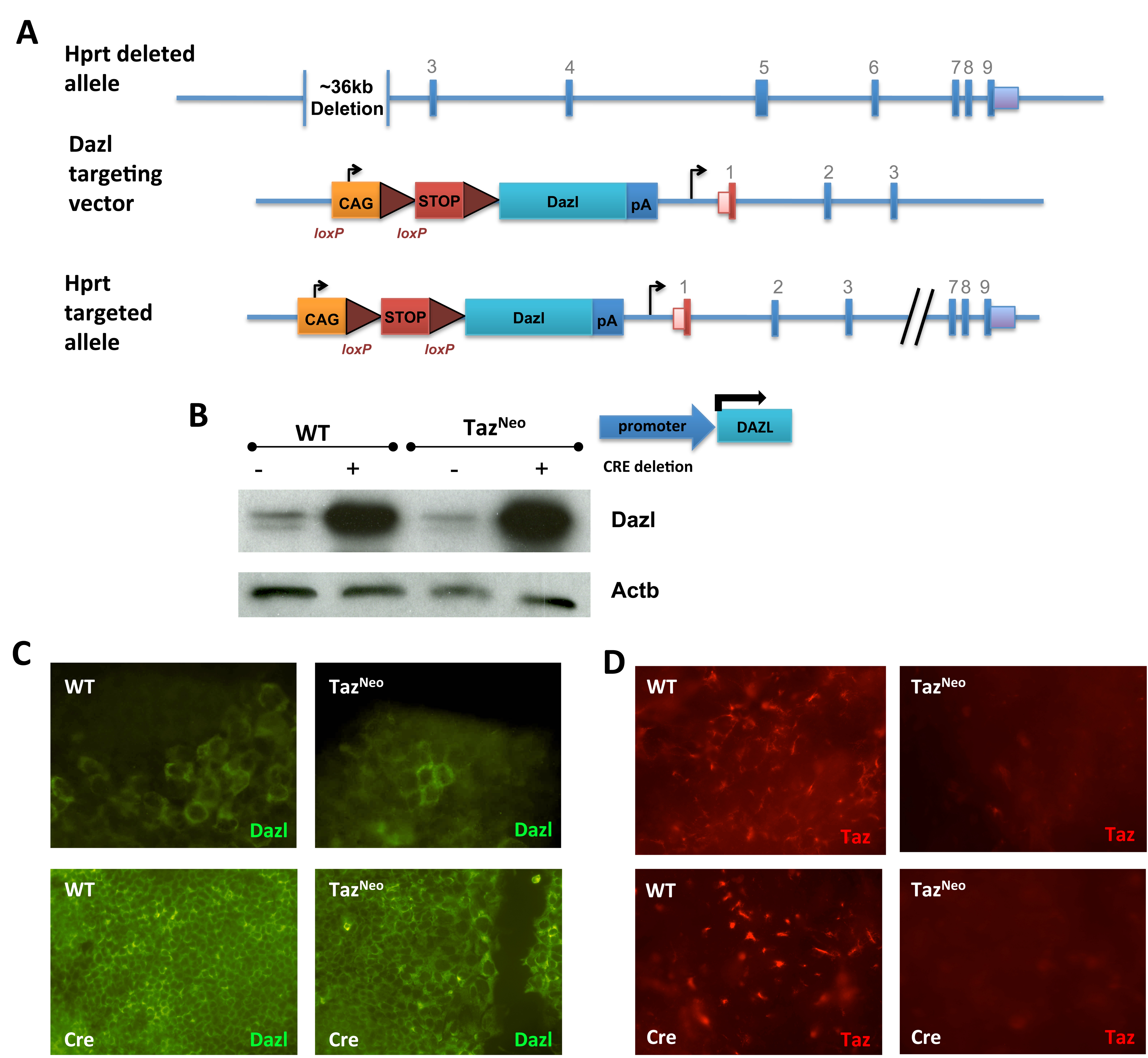

Supplement: S3 Fig — (A) Diagram of targeting vector used to over express Dazl in targeted ES cells (B) Western blot showing Dazl expression levels in wild-type (WT) and TazNeo targeted ES cells. Dazl (C) and Taz (D) immunostaining in WT (left panel) and Taz Neo (right panels) ES cells without (top panels) or with (bottom panels) CRE recombination. (TIF) [file pone.0131066.s003.tif]

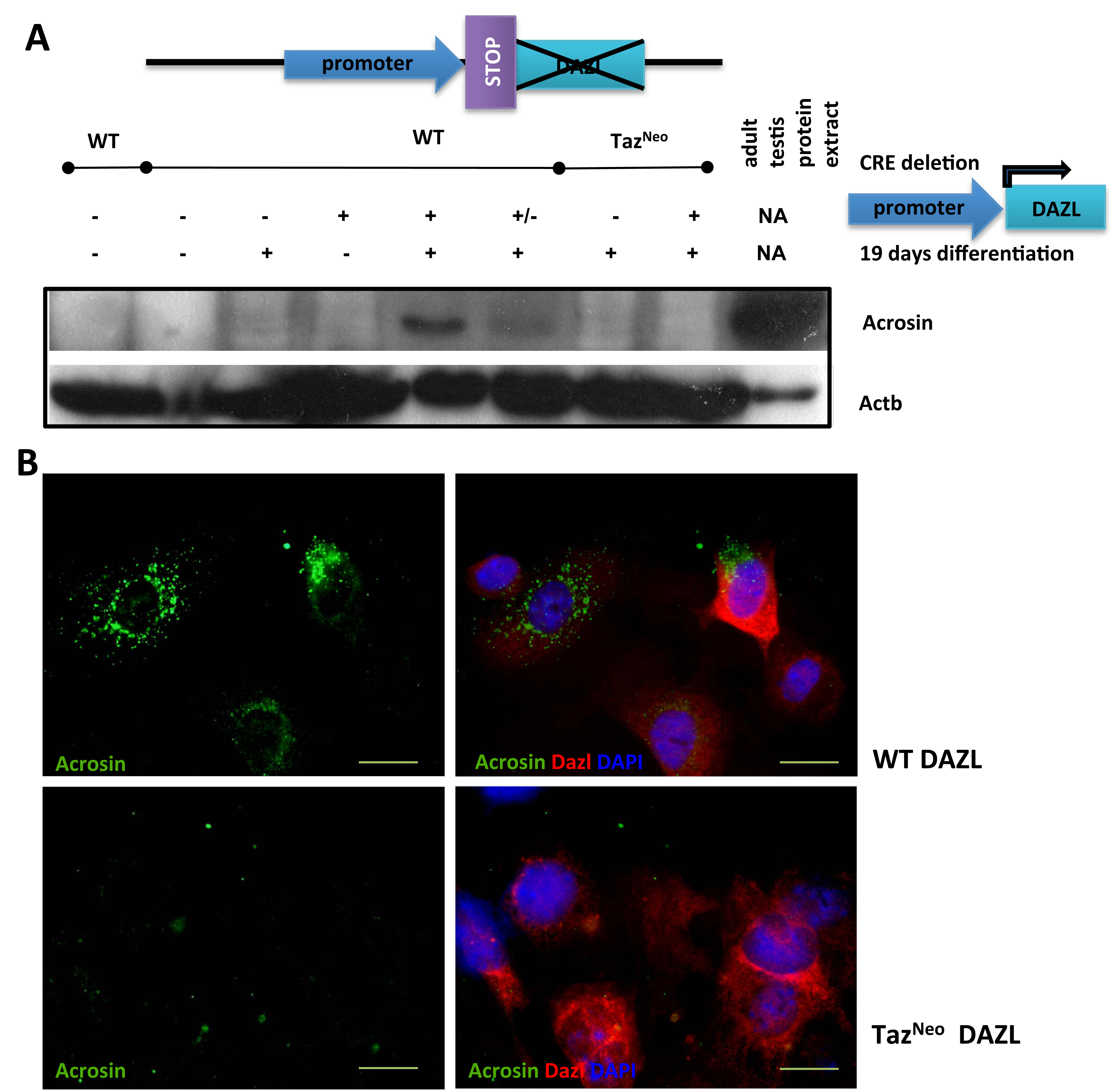

Supplement: S4 Fig — (A) Western blot of showing Acrosin expression in ES cells with or without Dazl expression following Cre deletion of the stop cassette and before or after 19 days of differentiation. The sample labelled +/- is a mixture of Dazl expressing and non-expressing cells. β-actin is used to assess equal loading. (B) Immunostaining of Acrosin (green) protein is expressed in fully differentiated Dazl (red) expressing parental HM1 ES cells (top panels) but not in Taz deficient clones (lower panels). The cells were differentiated for 19 days prior to immunostaining. Right panels show merged pictures of Acrosin (green), Dazl (red) and Dapi (blue). Scale bar: 50μm. (TIF) [file pone.0131066.s004.tif]
