# Supplementary material for: Mouse Tafazzin Is Required for Male Germ Cell Meiosis and Spermatogenesis
Source: PLoS One. 2015 Jun 26;10(6):e0131066. doi: 10.1371/journal.pone.0131066 (PMC4483168; doi:10.1371/journal.pone.0131066)
Supplement: S1 Table — (PDF) [file pone.0131066.s005.pdf]

S1 Table. Expression of marker genes used for staining Taz^{Neo} testis tubules

	Spermatogonia	Early Spermatocytes	Late Spermatocytes	Haploid Round Spermatids	Spermatozoa	Reference
Dazl	+	+				[1, 2]
Mvh (Ddx4)	+	+	+	+		[3, 4]
Actl7b				+		[5, 6]
Hook1					+	[7, 8]
PGK2					+	[9, 10]

1. Kee K, Angeles VT, Flores M, Nguyen HN, Reijo Pera RA. Human DAZL, DAZ and BOULE genes modulate primordial germ-cell and haploid gamete formation. Nature. 2009;462(7270):222-5. Epub 2009/10/30. doi: 10.1038/nature08562. PubMed PMID: 19865085; PubMed Central PMCID: PMC3133736.
2. Saunders PT, Turner JM, Ruggiu M, Taggart M, Burgoyne PS, Elliott D, et al. Absence of mDazl produces a final block on germ cell development at meiosis. Reproduction. 2003;126(5):589-97. Epub 2003/11/13. PubMed PMID: 14611631.
3. Noce T, Okamoto-Ito S, Tsunekawa N. Vasa homolog genes in mammalian germ cell development. Cell structure and function. 2001;26(3):131-6. Epub 2001/09/22. PubMed PMID: 11565805.
4. Tanaka SS, Toyooka Y, Akasu R, Katoh-Fukui Y, Nakahara Y, Suzuki R, et al. The mouse homolog of Drosophila Vasa is required for the development of male germ cells. Genes & development. 2000;14(7):841-53. Epub 2000/04/15. PubMed PMID: 10766740; PubMed Central PMCID: PMC316497.
5. Hisano M, Ohta H, Nishimune Y, Nozaki M. Methylation of CpG dinucleotides in the open reading frame of a testicular germ cell-specific intronless gene, Tact1/Actl7b, represses its expression in somatic cells. Nucleic acids research. 2003;31(16):4797-804. Epub 2003/08/09. PubMed PMID: 12907721; PubMed Central PMCID: PMC169926.
6. Chadwick BP, Mull J, Helbling LA, Gill S, Leyne M, Robbins CM, et al. Cloning, mapping, and expression of two novel actin genes, actin-like-7A (ACTL7A) and actin-like-7B (ACTL7B), from the familial dysautonomia candidate region on 9q31. Genomics. 1999;58(3):302-9. Epub 1999/06/22. doi: 10.1006/geno.1999.5848. PubMed PMID: 10373328.
7. Yamauchi Y, Ward MA. Preservation of ejaculated mouse spermatozoa from fertile C57BL/6 and infertile Hook1/Hook1 mice collected from the uteri of mated females. Biol Reprod. 2007;76(6):1002-8. Epub 2007/02/23. doi: 10.1095/biolreprod.106.059881. PubMed PMID: 17314312.
8. Mendoza-Lujambio I, Burfeind P, Dixkens C, Meinhardt A, Hoyer-Fender S, Engel W, et al. The Hook1 gene is non-functional in the abnormal spermatozoon head shape (azh) mutant mouse. Hum Mol Genet. 2002;11(14):1647-58. Epub 2002/06/21. PubMed PMID: 12075009.
9. Danshina PV, Geyer CB, Dai Q, Goulding EH, Willis WD, Kitto GB, et al. Phosphoglycerate kinase 2 (PGK2) is essential for sperm function and male fertility in mice. Biol Reprod. 2010;82(1):136-45. Epub 2009/09/18. doi: 10.1095/biolreprod.109.079699. PubMed PMID: 19759366; PubMed Central PMCID: PMC2802118.
10. Yoshioka H, Geyer CB, Hornecker JL, Patel KT, McCarrey JR. In vivo analysis of developmentally and evolutionarily dynamic protein-DNA interactions regulating transcription of the Pgk2 gene during mammalian spermatogenesis. Molecular and cellular biology. 2007;27(22):7871-85. Epub 2007/09/19. doi: 10.1128/MCB.00990-07. PubMed PMID: 17875925; PubMed Central PMCID: PMC2169153.
